# Introduction to the Special Issue: Halophytes in a changing world

**DOI:** 10.1093/aobpla/plv020

**Published:** 2015-05-06

**Authors:** Timothy J. Flowers, Adele Muscolo

**Affiliations:** 1 School of Life Sciences, University of Sussex, Falmer, Brighton BN1 9QG, UK; 2 Department GESAF, Agriculture Faculty, Mediterranea University, Feo di Vito, 89124 Reggio Calabria, Italy

**Keywords:** Climate change, halophyte, salinity, salt

## Abstract

Climate change will bring about rising sea levels and increasing drought, both of which will contribute to increasing salinization in many regions of the world. There will be consequent effects on our crops, which cannot withstand significant salinization. This Special Issue looks at the roles that can be played by halophytes, extremophiles that do tolerate salinities toxic to most plants. In an ecological context, papers deal with the conservation of a rare species, the effects of rising concentrations of CO_2_ and flooding on coastal vegetation, and the consequences of tree planting in inland plains for salinization. Physiological studies deal with the different effects of chlorides and sulfates on the growth of halophytes, the ability of some parasitic plants to develop succulence when growing on halophytic hosts and the interesting finding that halophytes growing in their natural habitat do not show signs of oxidative stress. Nevertheless, spraying with ascorbic acid can enhance ascorbic acid-dependent antioxidant enzymes and growth in a species of *Limonium*. Enzymes preventing oxidative stress are expressed constitutively as is the case with the vacuolar H-ATPase, a key enzyme in ion compartmentation. A comparison of salt-excreting and non-excreting grasses showed the former to have higher shoot to root Na^+^ ratios than the latter. A particularly tolerant turf grass is described, as is the significance of its ability to secrete ions. A study of 38 species showed the importance of the interaction of a low osmotic potential and cell wall properties in maintaining growth. From an applied point of view, the importance of identifying genotypes and selecting those best suited for the product required, optimizing the conditions necessary for germination and maximizing yield are described. The consequence of selection for agronomic traits on salt tolerance is evaluated, as is the use of halophytes as green manures. Halophytes are remarkable plants: they are rare in relation to the total number of flowering plants and they tolerate salinities that most species cannot. It is clear from the papers published in this Special Issue that research into halophytes has a distinct place in aiding our understanding of salt tolerance in plants, an understanding that is likely to be of importance as climate change and population growth combine to challenge our ability to feed the human population of the world.

## Introduction

By the year 2050, the world population is projected to stabilize at around 9.5 billion people (http://faostat3.fao.org/home/E). In order to feed this population adequately, global agriculture must double its food production and farm productivity must increase by 1.8 % each year—indeed a tall order. The challenges of attaining accelerated and sustainable growth and food security have been, and will likely continue to be, exacerbated by global climate change and extreme weather fluctuations. The main impacts of climate change are higher and more variable temperatures, changes in precipitation patterns (often lower and more erratic rainfall) and increasing soil salinity. Salinity is among the most widespread constraints in irrigated agriculture, affecting up to 7 % of the world's total land area and about one-third of irrigated land (see Flowers and Colmer 2008). Thus, salt tolerance is an agronomically important trait that requires attention among scientists worldwide. Despite technological advances, engineering a salt-tolerant plant is likely to take many years because many genes are involved. While those genes that are regulated under salt stress may be identified through the analysis of either RNA (Kawasaki *et al*. 2001) or proteins (Salekdeh *et al*. 2002), finding key genes for salinity tolerance and engineering them into, for example, important cereals in a coordinated manner is still far away (see also Panta *et al*. 2014). However, a glimmer of hope lies in the existence of some truly salt-tolerant plants, halophytes, plants that can survive in seawater salt concentrations.

Halophytes have long been recognized, but it was not until the 20th century that they were studied systematically (see Waisel 1972; Huchzermeyer and Flowers 2013). While we still have much to learn about these salt-tolerant plants, it is clear that more than one mechanism operates to generate tolerance—and hence the difficulties in engineering tolerance in more salt-sensitive species. Consequently, it is important to understand tolerance mechanisms operating at various levels, molecular, physiological and ecological, in order to develop an understanding of what is involved in being a halophyte. The long-term aim of such research is to be able to utilize knowledge of halophytes for improvement of the performance of crops in salt-affected soils. However, halophytes are not only valuable as scientific models, but also have potential as crops in their own right in saline agriculture (Flowers and Yeo 1995; Rozema and Flowers 2008; Panta *et al*. 2014), where they may be used for food, fibre and industrial purposes (Rozema *et al*. 2013). In this Special Issue, research papers and reviews address a variety of aspects of the biology of halophytes, in particular dealing with their ecology, physiology and potential as crops.

## Ecology and Conservation

Coastal areas are particularly vulnerable to some of the consequences of predicted changes in the climate associated with rising levels of atmospheric CO_2_. For instance, future increases in sea level and tidal surges are likely to lead to coastal inundation with seawater and consequent submergence of coastal wetlands. **Duarte, Santos, Silva, Marques, Caçador and Sleimi** report their investigations of underwater fluxes of O_2_ at different concentrations of dissolved CO_2_ for two halophytes common on coastal saltmarshes, *Halimione* (*Atriplex*) *portulacoides* and *Spartina maritima.* Photosynthesis was enhanced in both species for reasons that included reduction of oxygenase activity of Rubisco. The authors concluded that the role of these two species as primary producers in estuarine ecosystems will be reinforced in the future. Coastal areas and other saline environments are major contributors to regional and global biodiversity. In these environments, rapidly changing gradients require highly specialized plants like halophytes. **Caperta, Espirito-Santo, Silva, Ferreira, Paes, Róis, Costa and Arsénio** investigated the ecology and conservation of the halophyte *Limonium multiflorum*, endemic to the west coast of Portugal and listed in the IUCN red list of threatened species, in order to determine its habitat requirements and conservation status. Their work showed narrow habitat specificity for this species, which is intolerant of competition with invasive alien plants, suggesting that *in situ* conservation in a local ‘hotspot’ of this rare and vulnerable species is a priority in order to ensure that biodiversity is retained. **Tóth, Balog, Szabó, Pásztor, Jobbágy, Nosetto and Gribovszki** examined the consequence of tree planting in the Great Hungarian Plain, as an example of a flat sedimentary area with a sub-humid climate. Measurements of tree growth and soil and groundwater salinity showed that accumulated tree biomass was positively correlated with rates of soil salinization, although leaching of salts during the winter months mitigated overall salinization.

## Physiology

For halophytes, functional traits, those plant attributes that significantly influence establishment and survival, include any mechanisms that contribute to their tolerance of high soil or water salinity as well as other abiotic stresses of their habitats, such as drought or flooding. Papers in this Special Issue address the ecophysiological mechanisms of salinity tolerance in halophytes. **Reginato, Castagna, Furlán, Castro, Ranieri and Luna** used the halophytic shrub *Prosopis strombulifera* to investigate whether the ability of this species to grow under increasing concentrations of mixtures of Na_2_SO_4_ and NaCl was related to the synthesis of antioxidants that counter the generation of reactive oxygen species and/or maintain photosynthesis. They investigated whether the synthesis of polyphenolic compounds and the maintenance of leaf pigment contents contributed to tolerance. Their results showed a marked difference in response to the two salts. While Na_2_SO_4_ sharply increased antioxidants, NaCl did not affect H_2_O_2_ content, lipid peroxidation, pigments or polyphenol synthesis. They suggested a role for flavonoids in counteracting the oxidative damage induced by Na_2_SO_4_, showing that ionic interactions between different salts in salinized soils are able to modify the biochemical and morpho-physiological responses to salinity. **Gil, Bautista, Boscaiu, Lidón, Wankhade, Sánchez, Llinares and Vicente** included antioxidant activity in their study of changes in the contents of chemical markers associated with stress responses in five halophytes (*Sarcocornia fruticosa*, *Inula crithmoides*, *Plantago crassifolia*, *Juncus maritimus* and *J. acutus*) growing on a Mediterranean salt marsh. The authors showed that the tolerance mechanisms were constitutive and relatively independent of external conditions. No correlation was observed between the degree of environmental stress and the levels of internal oxidative stress assessed by formation of malondialdehyde or enzymatic and non-enzymatic antioxidants. Their data suggest that these halophytes were not subjected to oxidative stress under natural conditions and did not, therefore, need to activate antioxidant defence mechanisms. The main tolerance mechanisms of regulating Na^+^, Cl^−^ and osmolyte concentrations were constitutive. However, the ability of exogenous application of ascorbic acid to enhance the salt tolerance of *Limonium stocksii*, a halophyte from the sub-tropics, is described by **Hameed, Gulzar, Aziz, Hussain, Gul and Khan**. By comparison with plants sprayed with water, the authors showed that spraying with an aqueous solution of ascorbic acid enhanced growth, decreased the production of malondialdehyde and increased the activity of the antioxidant enzymes ascorbate peroxidase and glutathione reductase. The activities of other enzymes involved in antioxidant cycles were, however, unaffected by exogenous application of ascorbic acid.

The importance of ion compartmentation to salt tolerance lies behind the paper of **Katschnig, Jaarsma, Almeida, Rozema and Schat**, who investigated the transport activity of the tonoplast Na^+^/H^+^ antiporter, V-H^+^-ATPase and V-H^+^-PPase in a highly tolerant salt-accumulating halophyte *Salicornia dolichostachya*. Using isolated vesicles from the tonoplast they showed that activity of the V-H^+^-ATPase and V-H^+^-PPase in *S. dolichostachya* was not affected by salt treatment, again suggesting constitutive expression of the proteins involved. A high tonoplast H^+^ gradient at low external salinities is likely to have contributed to the high cellular salt accumulation of this species. The ability of a mistletoe *Plicosepalus acaciae* to accumulate Na^+^ and Cl^−^ when growing on halophytic hosts in the Jordan valley was investigated by **Veste, Todt and Breckle**. By comparing plants collected from the wild and growing on either halophytic or non-halophytic hosts, the authors were able to show that those growing on halophytes had three-times higher water content than plants on non-halophytes, with increased ion concentrations and four to five times higher leaf volumes. They concluded that *P. acaciae* could be described as a facultative euhalophyte whose xylem sap concentrations of Na^+^ and Cl^−^ reflected that of the host. While compartmentalizing ions in leaves is a vital aspect of salt tolerance in all halophytes, some are also able to excrete ions. **Moinuddin, Gulzar, Ahmed, Gul, Koyro and Khan** identified differential adaptive response patterns of salt-excreting (*Aeluropus lagopoides* and *Sporobolus tremulus*) versus non-excreting (*Paspalum paspalodes* and *Paspalidium geminatum*) halophytic grasses grown under non-saline and saline conditions. They showed that growth and relative growth rate decreased under saline conditions in the order *P. geminatum* > *S. tremulus* = *A. lagopoides* > *P. paspalodes*. The salt-secreting grasses had a higher shoot : root Na^+^ ratio than non-excreting grasses, and the high salt resistance of *P. paspalodes* was attributed to a lower Na^+^ uptake, higher nitrogen-use efficiency and higher water-use efficiency amongst the grasses investigated. **Tada, Komatsubara and Kurusu** also investigated a salt-tolerant grass. They identified a genotype of *Sporobolus virginicus*, a halophytic turf grass collected in Japan, and investigated its growth rate, ion relations and proline concentration in comparison with the reported properties of genotypes collected from the USA, South Africa and Egypt. Surprisingly, the Japanese genotype showed a salinity tolerance up to 1.5 M NaCl, with shoot and root growth being stimulated by salt, an unusual feature amongst halophytic grasses. Plants accumulated Na^+^ and CI^−^ in shoots and roots in the presence of external salinity but K^+^ was accumulated to a higher level than reported in other genotypes of *S. virginicus*, resulting in a relatively high K^+^/Na^+^ ratio. Under salinity stress, plants accumulated proline in proportion to the NaCl concentration. Cultured cells also accumulated proline, an osmolyte that likely plays an important role in plant–water relations at the cellular and whole plant level. Osmotic adjustment, however, involves not only the uptake and accumulation of osmotically active compounds, but also changes in wall properties and **Touchette, Marcus and Adams** studied the flexibility of leaf tissues of 38 freshwater, coastal and marine (seagrasses and salt marsh) plant species. Their results, comparing the modulus of elasticity and changes in osmotic potential, show that leaf tissues of plants acclimated to marine environments tend to have higher elasticity and lower osmotic potential than freshwater plants in agreement with the general tenets of the cell water conservation hypothesis that elevated elasticity and comparatively low osmotic potential work in concert to maintain vital cellular water content.

## Crops

Apart from their value as models of salt tolerance, halophytes are also a source of secondary metabolites with potential economic value. The steady-state pools of many stress-related metabolites are already enhanced in halophytes as compared with glycophytes, but growth in conditions away from the optimum can induce further changes to secondary metabolites such as antioxidants. **Boestfleisch, Wagenseil, Buhmann, Seal, Wade, Muscolo and Papenbrock** demonstrated that by altering the salinity of the growing environment it is possible to alter the concentration of total phenols, flavonoids, ascorbate, reduced/oxidized glutathione and ROS scavenging enzymes in seedlings and plants from halophytes of different families (Amaranthaceae, Brassicaceae, Plantaginaceae and Rhizophoraceae). They identified *Tripolium pannonicum*, *Plantago coronopus*, *Lepidiumlatifolium* and *Salicornia europaea* as having most potential as functional foods or for the supply of nutraceuticals. **Singh, Buhmann, Flowers, Seal and Papenbrock** report further investigations into the choice of species of *Salicornia* and *Sarcocornia* as potential crops. They collected seed from Germany, Israel, Kazakhstan, the Netherlands and the UK, for which they analysed the External Transcribed Spacer (ETS) sequence. ETS sequence data clearly separated the two genera but not all of the species of *Salicornia*. The optimal growth conditions for *S. dolichostachya* included 100 mM NaCl in the culture medium and repeated harvesting was possible as a means to increasing yield. **Panuccio, Jacobsen, Akhtar and Muscolo** investigated the potential of a facultative halophytic species, quinoa, with a high protein content and unique amino acid composition as a new crop that might be cultivated in the presence of seawater or different salts. The results showed that at low concentrations all salts investigated increased the germination rate but not the germination percentages. An efficient antioxidant mechanism was present in quinoa, activated by salts during germination and early seedling growth, and osmotic and ionic stress factors had different degrees of influence on germination and development. **Rozema, Cornelisse, Zhang, Li, Bruning, Katschnig, Broekman, Ji and van Bodegom** looked at another well-established crop, sugar beet, and compared its tolerance with its wild halophytic relative, sea beet. In hydroponic experiments in which relative growth rate and its components were measured, they found that breeding for traits that lead to a high sugar content had only slightly reduced the salt tolerance of sugar beet relative to sea beet. **Ventura, Myrzabayeva, Alikulov, Omarov, Khozin-Goldberg and Sagi** focussed their study on different genotypes of *Crithmum maritimum* (originating from France, Portugal and Israel), a halophytic plant naturally found on rocky coastlines of the Atlantic Ocean and the Mediterranean Sea, that could be used as a cash crop for biosaline agriculture. Their results highlight variations among genotypes from different origins in salt-induced changes in plant growth, flowering behaviour and leaf metabolites with nutritional value such as ascorbic acid, suggesting that genotypic characteristics should be taken into account when evaluating a wild plant species for future crop cultivation. Finally, **Bruning, van Logtestijn, Broekman, de Vos, Parra González and Rozema** showed that when a legume *Melilotus officinalis* was grown in a saline field in the Netherlands, virtually all of its nitrogen was derived from symbiotic fixation and advocated its use as a green manure in temperate saline agriculture.

## Conclusions

Halophytes are remarkable plants. They are rare in relation to the total number of flowering plants and they tolerate salinities that most species cannot, yet these salinities are widespread on the planet. It is clear from the papers published in this Special Issue that research into halophytes has a distinct place in aiding our understanding of salt tolerance in plants, which is likely to be important as climate change and population growth combine to challenge our ability to feed the human population of the world. Studying halophytes will provide basic information on salt tolerance, aiding the transformation of current crops as well as providing new halophytic crops.

## Conflict of Interest Statement

None declared.
